# Dietary Potassium Attenuates the Effects of Dietary Sodium on Vascular Function in Salt-Resistant Adults

**DOI:** 10.3390/nu12051206

**Published:** 2020-04-25

**Authors:** Katarina Smiljanec, Alexis Mbakwe, Macarena Ramos Gonzalez, William B. Farquhar, Shannon L. Lennon

**Affiliations:** Department of Kinesiology and Applied Physiology, University of Delaware, Newark, DE 19716, USA; ksmilja@udel.edu (K.S.); ambakwe@udel.edu (A.M.); macramos@udel.edu (M.R.G.); wbf@udel.edu (W.B.F.)

**Keywords:** Potassium, sodium, endothelium, vascular function

## Abstract

The influence of dietary sodium and potassium on blood pressure (BP) has been extensively studied, however their impact on endothelial function, particularly any interactive effects, has received less attention. The purpose of this study was to determine if dietary potassium can offset the deleterious effect of high dietary sodium on endothelial function independent of BP. Thirty-three adults with salt-resistant BP (16 M and 17 F; 27 ± 1 year) completed seven days each of the following diets in a random order: a moderate potassium/low sodium diet (65 mmol potassium/50 mmol sodium; MK/LS), a moderate potassium/high sodium diet (65mmol potassium/300 mmol sodium; MK/HS) and a high potassium/high sodium (120 mmol potassium/300 mmol sodium; HK/HS). On day seven of each diet, 24-h ambulatory BP and a urine collection were performed. Brachial artery flow-mediated dilation (FMD) was measured in response to reactive hyperemia. Between diets, 24-h BP was unchanged confirming salt resistance (*p* > 0.05). Sodium excretion increased on both HS diets compared to MK/LS (*p* < 0.05) and potassium excretion was increased on the HK diet compared to MK/LS and MK/HS (*p* < 0.05) confirming diet compliance. FMD was lower in MK/HS (5.4 ± 0.5%) compared to MK/LS (6.7 ± 0.5%; *p* < 0.05) and HK/HS (6.4 ± 0.5%), while there was no difference between the MK/LS and HK/HS diets (*p* > 0.05). These data suggest that dietary potassium provides vascular protection against the deleterious effects of high dietary sodium by restoring conduit artery function.

## 1. Introduction

Cardiovascular disease remains a major public health problem in the U.S. [1] and is the result of various risk factors including lifestyle factors such as nutrition. Both dietary sodium and potassium are notable for their blood pressure (BP) raising and lowering capabilities, respectively [2,3,4,5]. In that regard, the average sodium consumption is consistently above the intake recommended by the Dietary Guidelines for Americans and organizations such as the American Heart Association [1,6]. A recent change in the potassium guidelines [7] suggests that adult women are meeting the guidelines, but intake in men remains low. While the effect of these two nutrients on BP is fairly well understood, their impact on vascular function, particularly any interactive effects, has received less attention. Endothelial dysfunction, characterized by impaired dilation, is an important non-traditional risk factor for atherosclerosis [8]. Brachial artery flow-mediated dilation (FMD) has been used to assess endothelial function non-invasively and can be useful in predicting risk of future cardiovascular events [9].

The addition of a high potassium diet, such as the Dietary Approaches to Stop Hypertension (DASH) diet, to high sodium consumption has successfully lowered BP in adults with pre-hypertension and hypertension [2] demonstrating potassium’s BP lowering abilities. While randomized controlled trials have demonstrated the beneficial effects of dietary potassium on BP [4,5], there is emerging evidence that a diet high in potassium may be beneficial to the health of the vasculature separate from its role on BP. Indeed, salt-sensitive rodents supplemented with potassium while consuming a high salt chow were protected against vascular injury [10] and had improved left ventricular relaxation as assessed by echocardiography [11]. We have previously shown that chronic high dietary sodium intake has detrimental effects on the vasculature, independent of or before any changes in BP in salt-resistant adults [12,13]. To date, studies that have evaluated the role of potassium on sodium have been acute and focused on the post-prandial changes in endothelial function. These studies have shown an attenuation of the post-meal reduction in endothelial function [14]. However, it is unknown if chronic dietary potassium intake can lessen the effect of high dietary sodium on the vasculature independent of BP changes. Therefore, the purpose of this investigation was to test the hypothesis that a diet high in potassium can attenuate the deleterious effects of a high sodium diet on endothelial function in salt-resistant adults. 

## 2. Materials and Methods 

### 2.1. Study Population

Thirty-three healthy salt-resistant individuals aged 22–45 participated in this study. A total of 63 individuals were screened. Two were excluded for not meeting the inclusion criteria and 17 dropped out for various reasons including a lack of time or continued interest. Eleven were excluded due to missing data collection visits, non-compliance with urine collection, or lack of FMD data. None were excluded due to salt sensitivity status. The study protocol was approved by the Institutional Review Board of the University of Delaware and conforms to all of the provisions of the Declaration of Helsinki. Verbal and written consent was obtained from all subjects prior to enrollment in the study. This study is registered on ClinicalTrials.gov (NCT03265353). 

### 2.2. Experimental Protocol

Subjects reported to our nurse managed primary care center on the University of Delaware campus for a screening visit following a 12 h fast. The screening visit included a general physical exam and assessment of past medical history. During this visit, a venous blood sample was also collected. Subjects with a history of hypertension, cardiovascular disease, malignant cancer, diabetes mellitus, or renal disease were excluded. Subjects with a body mass index (BMI) of 30 kg/m^2^ or greater, those who used tobacco products, and post-menopausal women were excluded. Post-menopausal women were excluded because salt sensitivity of BP increases with menopause and endothelial function declines [15]. 

### 2.3. Dietary Potassium and Sodium Manipulation 

This research project was a controlled feeding study with all food prepared by a registered dietitian. All subjects consumed three diets, each seven days in length in a random order. The three diets were a moderate potassium/low sodium diet containing 65 mmol potassium/50 mmol sodium (MK/LS); a moderate potassium/high sodium diet containing 65 mmol potassium/300 mmol sodium (MK/HS); and a high potassium/high sodium diet containing 120 mmol potassium/300 mmol sodium (HK/HS). The sodium intakes were selected in order to allow us to accurately classify adults with salt-resistant BP and are in agreement with previously published studies [12,16]. The moderate potassium intake mimicked the average potassium intake in the U.S. [17] and the high potassium diet met the 2005 Dietary Reference Intake guidelines [18]. The Mifflin-St. Jeor equation was used to adjust the energy content of the diet to maintain a constant body weight [19]. The controlled diets were designed to contain the same amount of the macronutrients. Each diet consisted of approximately 50% carbohydrates, 30% fat, and 20% protein. Daily fluid intake was monitored and recorded. Subjects were instructed to maintain normal physical activity levels throughout the study.

### 2.4. Twenty-four Urine and Blood Pressure 

A 24-h urine collection was conducted on the last day of each diet. Urine was analyzed for total volume, urinary electrolytes (Easy-Electrolyte Analyzer; Medica, Bedford, MA, USA), and urine osmolality (Advanced 3D3 Osmometer; Advanced Instruments, Norwood, MA, USA). Free water clearance and fractional excretion of sodium and chloride were calculated using standard equations. Urine collections were considered incomplete if collections occurred outside the 20–28 h timeframe, if there were two or more missed collections, or if total volume was less than 500 mL. During the same 24-h period, subjects also wore an ambulatory BP monitor (Oscar 2; SunTech Medical, Morrisville, NC, USA) on their arm. BP was measured every 20 min while the subject was awake and every 30 min during sleep. This device has been validated for brachial BP measurements [20]. A subject was considered adherent if at least 75% the readings were successful [21]. Laboratory BP was also measured by an automated oscillometric sphygmomanometer (Dash 2000; GE Medical Systems, Milwaukee, WI, USA) during the experimental visits. 

### 2.5. Salt Resistance Classification

Salt resistance was defined as a change of 5 mmHg or less in 24-h mean arterial pressure (MAP) [22], and was determined while on the MK/LS and MK/HS diets. In both these diets, potassium is similar while the sodium content is purposely high and low to evaluate a potential change. This assessment is reproducible (>90%) within participants who are either normotensive or hypertensive [23,24,25,26] and was defined on an individual basis after completion of the entire protocol. 

### 2.6. Blood Markers

Hemoglobin (Hb 201+ model; HemoCue, Lake Forest, CA, USA), hematocrit (Sorvall Legend Micro 17 Microcentrifuge with Microhematocrit Reader; Thermo Scientific, Waltham, MA, USA), serum electrolytes (EasyElectrolyte Analyzer; Medica, Bedford, MA, USA), and plasma osmolality (Advanced 3D3 Osmometer; Advanced Instruments, Norwood, MA, USA) were measured from a venous blood sample obtained during each experimental visit. 

Plasma renin activity (PRA), serum angiotensin II, and plasma aldosterone were also measured from a venous blood sample for each visit via radioimmunoassay by the Biomarker Analytical Core at Wake Forest University Baptist Medical Center. 

### 2.7. Vascular Measures

#### Assessment of Brachial Artery Flow-Mediated Dilation (FMD)

Brachial artery flow-mediated dilation (FMD) was assessed according to established guidelines [9]. Subjects were assessed on the final day of each diet. Subjects were supine with their right arm supported at heart level. A BP cuff was placed on the proximal forearm approximately 3 cm below the antecubital crease. Longitudinal images of the brachial artery and continuous Doppler blood velocity were obtained using a 12 MHz linear phased array ultrasound transducer (GE P5; GE Healthcare, Waukesha, WI, USA). Following 20 min of rest, baseline images were recorded and blood velocity was obtained. The cuff was then rapidly inflated to 200 mmHg for 5 min. Images and blood velocity were recorded throughout this inflation period and continued for 2 min following cuff release to determine peak diameter change and to calculate shear rate. 

Ultrasound images were transmitted to a National Instruments IMAQ PCI-1411 image acquisition board at a frequency of 30 frames/s by way of an S-Video connection. Brachial artery diameter was determined using custom-designed automated edge detection software using National Instruments LabVIEW 8.0. Peak diameter was determined after applying a 3 s wide median filter to each data point. Reproducibility in our lab for this technique is 1.3 ± 1.1% and 1.9 ± 1.6% (coefficient of variation) for baseline and peak brachial diameters respectively. FMD was expressed as a % change from baseline, and Doppler blood velocity and diameter data were used to calculate shear rate area under the curve from cuff deflation to peak diameter. Shear rate area under the curve (AUC) has been shown to best represent the stimulus for dilation [27].

### 2.8. Pulse Wave Analysis 

A central aortic pressure wave was synthesized from the measured brachial artery pressure wave with the SphygmoCor XCEL system (AtCor Medical; Sydney, Australia), which uses a transfer function and is FDA approved. Central pressures and augmentation index (AIx) were obtained from the synthesized wave. AIx is an index of wave reflection and is influenced by arterial stiffness. AIx is calculated as the ratio between augmented pressure and central pulse pressure, or AIx = (P_2_–P_1_)/(P_s_–P_d_), where P_1_ is first shoulder of systolic pressure, P_2_ is second shoulder of systolic, P_s_ is peak systolic pressure, and P_d_ is end-diastolic pressure. Measures were performed in triplicate. 

### 2.9. Pulse Wave Velocity 

Carotid-femoral pulse wave velocity (PWV) was measured using applanation tonometry and the same Sphygmocor XCEL system as above while the subject was at rest in a supine position. Carotid and femoral pressure waveforms were recorded simultaneously using a high-fidelity strain-gauge transducer (Millar Instruments; Houston, TX, USA) placed over the carotid artery and a BP cuff placed on the upper thigh, respectively. PWV distance was measured using the subtraction method where proximal distance (carotid measurement site to the sternal notch) was subtracted from distal distance (sternal notch to the thigh cuff). Carotid-femoral PWV was calculated by dividing the measured aortic distance (distal–proximal) by the average measured time delay between the initial upstrokes of corresponding carotid and femoral waveforms. Measurements were performed in duplicate. 

### 2.10. Statistical Analysis 

The primary outcome was brachial artery FMD. A repeated-measures ANOVA was performed between the three diets for all vascular measures, urinary sodium and potassium excretion, and hormones. Post hoc tests were conducted when appropriate. Data was analyzed using IBM SPPS Statistics for Windows, version 26.0 (IBM Corp.; Armonk, NY, USA). Data are represented as mean ± standard error of measurement (SE).

## 3. Results

### 3.1. Subject Characteristics

Baseline subject characteristics are provided in Table 1. There was a near even distribution between men and women in this study. Subjects were healthy, non-obese, and had normal blood pressure. Biochemical data highlighted normal fasting blood glucose, lipid panel, serum electrolytes, and renal function. 

### 3.2. Dietary Potassium and Sodium Manipulation

Hemodynamic and renal responses to the dietary manipulations are presented in Table 2. The high sodium diet increased serum sodium on the MK/HS but not the HK/HS, while serum chloride was elevated on both high sodium diets. In contrast, the high potassium diet did not alter serum potassium levels. Urine osmolality was elevated on both high sodium diets, while free water was negative, highlighting a more concentrated urine. Urine flow rate was increased on the HK/HS alone. 

**Figure 1:**
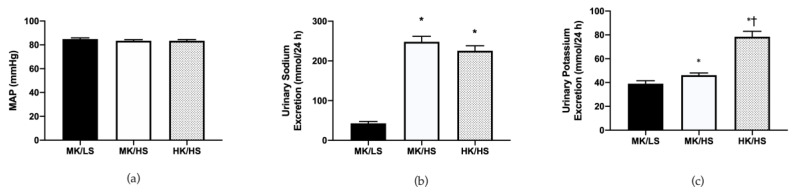
(**a**) Mean arterial pressure (MAP) on the last day of each diet. (**b**) Urinary sodium excretion increased on the high sodium diets compared to low sodium. (**c**) Urinary potassium excretion was elevated on the high potassium diet compared to the moderate potassium diets, however there was a difference in potassium excretion between the high and low sodium diets. HK/HS, high potassium/high sodium; MK/HS, moderate potassium/high sodium; MK/LS, moderate potassium/low sodium. Values are mean ± SE; * *p* < 0.05 vs. MK/LS; † *p* < 0.05 vs. MK/HS.

Consistent with our design, subjects were salt-resistant as there was no significant difference between MAP on the MK/LS and MK/HS diets (85 ± 1, 84 ± 1; *p* > 0.05) as shown in Figure 1. On the high sodium diets, 24-h urinary sodium excretion was significantly elevated, while 24-h potassium excretion was elevated on the high potassium diet. These data demonstrate that subjects were compliant with the controlled feeding study. 

**Figure 2:**
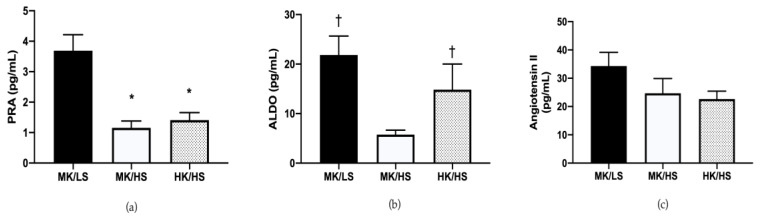
(**a**) Plasma renin activity (PRA) was suppressed on the high sodium diets. (**b**) Aldosterone (ALDO) levels were elevated on the MK/LS and HK/HS diets relative to MK/HS. (**c**) Angiotensin II levels were not significantly different between the three diets. HK/HS, high potassium/high sodium; MK/HS, moderate potassium/high sodium; MK/LS, moderate potassium/low sodium. Values are mean ± SE. * *p* < 0.05 vs. MK/LS; † *p* < 0.05 vs. MK/HS.

Figure 2 presents the hormone data from the three diets. As expected, the high sodium diets significantly suppressed PRA compared to low sodium. The aldosterone response was consistent with previous reports between the HS and LS diets combined with the moderate potassium diet, however the HK/HS fell in between. Plasma angiotensin II was greatest on the MK/LS diet however this did not reach statistical significance. 

### 3.3. Vascular Function

**Figure 3:**
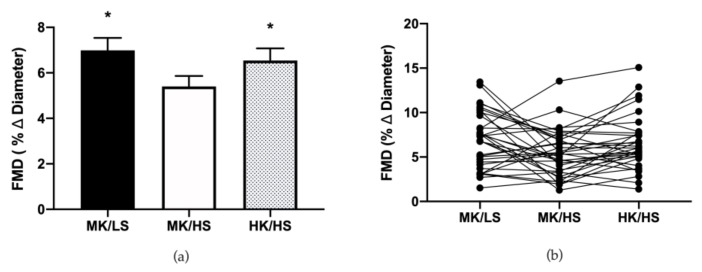
(**a**) Group brachial artery flow-mediated dilation (FMD) responses during the three diets. (**b**) Individual FMD responses during the three diets. Values are mean ± SE. FMD, flow-mediated dilation; HK/HS, high potassium/high sodium; MK/HS, moderate potassium/high sodium; MK/LS, moderate potassium/low sodium. * *p* < 0.05 vs. MK/HS.

Vascular function was assessed utilizing FMD to determine the effect of dietary manipulation of sodium and potassium on dilation of the brachial artery. Baseline and peak diameters for the brachial artery are shown in Table 3. There was no significant difference between these diameters nor AUC shear rate. FMD was reduced by approximately 23% when moving from the low to high sodium diet. This was rescued by inclusion of the high potassium diet (see Figure 3). 

**Figure 4:**
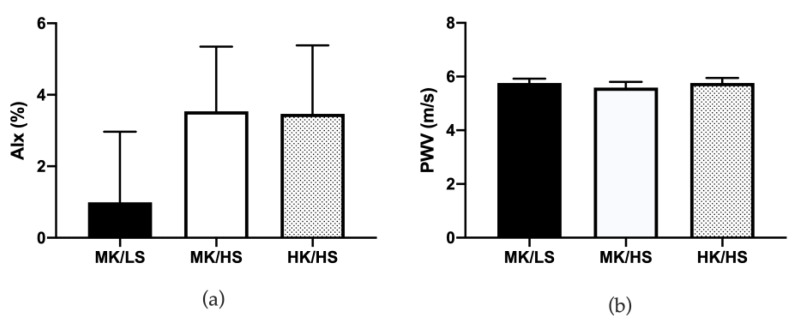
(**a**) Augmentation index (AIx) was unaltered over the three diets. (**b**) Pulse wave velocity (PWV) remained unchanged over the three diets. Values are mean ± SE. HK/HS, high potassium/high sodium; MK/HS, moderate potassium/high sodium; MK/LS, moderate potassium/low sodium.

AIx, an estimate of wave reflection and PWV, and a marker of arterial stiffness were unaltered across the three diets as shown in Figure 4.

## 4. Discussion

The major finding of this study is that a diet rich in potassium can attenuate the deleterious effects of high dietary sodium on endothelial function in salt-resistant adults. Furthermore, there was no difference in endothelial function between the MK/LS and HK/HS diets, suggesting that potassium can offset the effects of sodium on vascular function and this dietary pattern results in similar effects on the vasculature as a low sodium diet. Potassium is notable for its BP lowering effects in those with pre-hypertension and hypertension and in the presence of an elevated sodium intake [2,5,28]. Our group has previously demonstrated that a high sodium diet has BP independent effects on endothelial function [12,13]. What remained unknown however, was whether potassium could attenuate the effects of dietary sodium in the absence of a change in BP in salt-resistant adults. Our data highlight that a diet rich in potassium dampens sodium’s deleterious effects on the vasculature. 

Endothelial dysfunction is an independent, non-traditional risk factor for atherosclerosis [29] and often precedes the development of cardiovascular disease [8]. Dietary sodium has been highlighted as a dietary factor that increases the risk of high BP and subsequent forms of cardiovascular disease. Seven days of a high sodium diet decreased brachial artery FMD compared to a low sodium diet in salt-resistant adults [12], suggesting that sodium has BP independent effects on the vasculature. This is important as most young to middle-aged adults have a normal BP but consume a diet rich in sodium, which is potentially damaging their vasculature that is not evident by a change in BP. Furthermore, epidemiological studies highlight that most adults become hypertensive independent of salt sensitivity, suggesting that investigations in salt-resistant adults is important [30]. 

Evidence of potassium’s ability to lower sodium-induced elevations in BP is well described in the literature [2,5,28,31]. An inverse association between potassium and BP has been shown in those individuals consuming greater than 6 g salt/day [32]. Furthermore, potassium is beneficial to the vasculature separate from its role on BP [10,33,34,35]. Salt-sensitive rodents supplemented with potassium while consuming a high salt chow were protected against vascular injury [10] and demonstrated improved left ventricular relaxation as assessed by echocardiography [11]. In regard to human studies, several acute interventions have shown potassium to have favorable vascular effects [14,36,37]. A high potassium meal improved FMD two hours post-prandially compared to a low potassium meal [37]. When a high sodium meal was accompanied by a high potassium intake, reductions in post-prandial FMD were attenuated in healthy, normotensive adults suggesting that potassium could acutely dampen sodium’s reduction on FMD [14]. This was also shown after six days of a low versus high potassium diet on fasting FMD [36]. These data highlight a positive role for a high potassium intake on the vasculature, but this was not studied in the context of salt resistance. While BP did not change in several of these studies, this was only evaluated in the laboratory and not utilizing 24-h ambulatory BP monitoring. Our data clearly highlight the BP-independent effects of potassium on a high sodium diet in salt-resistant adults as mean arterial pressure did not change. 

Our hormone data support a role for dietary potassium as aldosterone was elevated on the HK/HS diet similarly to MK/LS and suppressed on the MK/HS diet alone. While serum potassium was not significantly elevated, there is evidence to suggest that increases in dietary potassium may cause a feedforward mechanism by the gut to the kidney to increase potassium excretion without changes in serum potassium [38,39]. The significant elevation of PRA on the low sodium diet responded as expected and as previously demonstrated [12,40]. The response of Ang II was variable, but the pattern of response was as expected. There is likely individual variability in response to the diets. These data suggest more work is needed to tease out relations between hormonal responses and mixed potassium diets.

While we observed improvements in brachial artery FMD on the HK/HS diet compared to MK/HS, we did not observe any change in AIx or PWV. AIx appears to be lower on the MK/LS compared to the two high sodium diets but this was not statistically significant, even when corrected for heart rate. Other studies report no change in AIx with potassium supplementation or a potassium rich diet [41,42,43]. Seven days is likely not long enough to elicit a change in AIx or PWV [44]. We have previously shown that dietary potassium excretion across a wide intake range correlates with improved PWV in young healthy adults [45], but in this study our potassium intake was held to two levels likely diminishing our ability to see this relation. This is in contrast to Berry et al. [43] who found no change in PWV after six weeks when the diet was supplemented with 20 or 40 mmol/d of potassium from fruit and vegetables or 40 mmol/d from a potassium citrate supplement. However, other supplementation studies have successfully lowered PWV. He et al. [46] improved PWV following 64 mmol/d of potassium bicarbonate or potassium chloride compared to a placebo in mild hypertensives. A similar finding was reported in individuals at risk for cardiovascular disease who consumed 64 mmol/d of potassium chloride [41]. Therefore, potassium may play a role in improving PWV in those with increased cardiovascular disease risk.

The mechanism responsible for potassium’s effect on endothelial function is not known. The mechanisms underlying sodium’s impact on endothelial dysfunction are still being explored. However, sodium-induced impairments in endothelial function have, in part, been attributed to increases in reactive oxygen species (ROS) [47,48,49,50]. An increase in ROS has been shown with salt loading [49] specifically leading to an increase in superoxide [31,51]. This increase in superoxide is thought to reduce the bioavailability and/or production of nitric oxide (NO), a potent vasodilator, by forming peroxynitrite, a potent oxidant [52]. Work in humans has established a role for sodium-induced oxidative stress. Greaney et al. [53] demonstrated improved cutaneous microvascular function with local ascorbic acid infusion under high dietary sodium conditions, while Ramick et al. [54] highlighted that the source of these radicals may be nicotinamide adenine dinucleotide phosphate (NADPH) oxidase, as cutaneous vasodilation was restored with local infusion of apocynin. Furthermore, endothelial cell nitrotyrosine content was increased on the high sodium diet. To date, not much is known about potassium’s ability to lower oxidative stress levels under high dietary sodium conditions. In an animal model, a high potassium diet lowered oxidative stress levels in spontaneously hypertensive rats fed a high sodium diet [33]. Hypertensive individuals infused with potassium chloride, thereby increasing extracellular potassium, demonstrated improved endothelium-dependent vasodilation as assessed by changes in forearm blood flow [55]. This has not been studied in normotensive salt-resistant adults and therefore, is an area for future investigation.

It may be that potassium protects the endothelium via non-ROS mechanisms. Stroke prone hypertensive rats supplemented with potassium had reduced intimal lesions and improved endothelial-dependent relaxation in response to acetylcholine despite no change in BP suggesting that potassium was protective of the endothelium [35]. The authors attributed this to a preservation of NO, as no differences were seen in response to sodium nitroprusside, a measure of endothelial-independent dilation. Similar findings have been shown in patients with essential hypertension. Potassium chloride was infused, and forearm blood flow evaluated, using strain gauge plethysmography. Potassium chloride had a vasodilating effect in the hypertensives with no change in controls while co-infusion of *N^G^*-monomethyl L-arginine (L-NMMA), an inhibitor of NO synthase, blunted this response [55]. Again, no differences in response to sodium nitroprusside were seen. While these data suggest that potassium facilitates endothelial-dependent dilation via the NO pathway, the mechanism remains unclear. More recent work has suggested that attenuation of enhanced sympathetic vasoconstriction may be the mechanism by which potassium protects the endothelium, although this was only present in younger Dahl salt-sensitive rats and not mature rats, and potassium did lower BP in this study [56]. Taken together, there may be multiple mechanisms by which potassium protects against high dietary sodium and the current research suggests this is through a preservation of NO.

There are a few limitations to this study. The goal of our study was to determine whether a high potassium diet may protect the vasculature in the presence of high sodium, therefore we did not include a high potassium/low sodium diet group. It is possible that a high potassium/low sodium diet would be more beneficial for vascular function as compared to the MK/LS and HK/HS diets. We did not assess oxidative stress in this study, therefore we cannot say whether potassium’s protective effects relate to a lowering of sodium-induced oxidative stress levels. Furthermore, our cohort was healthy and relatively young with a mean age of 33 and therefore, we cannot say how middle-age and older adults would respond. 

## 5. Conclusions

In conclusion, this study highlights dietary potassium’s protective effects on the vasculature in the presence of a high sodium diet. We extend previous findings demonstrating that dietary potassium is beneficial acutely on a high salt meal. Furthermore, we demonstrated these effects in salt-resistant adults using a controlled feeding study approach. These findings suggest that dietary potassium can be beneficial to those who consume a high sodium diet by protecting the endothelium. 

## Figures and Tables

**Table 1:** Baseline subject characteristics.

Baseline Characteristic	All Subjects
Demographic Data	
*N* (M/F)	33 (16/17)
Age (year)	27 ± 1
Height (cm)	172 ± 1.6
Mass (kg)	72 ± 1.8
Body Mass Index (kg/m^2^)	24 ± 0.5
Systolic BP (mmHg)	113 ± 3
Diastolic BP (mmHg)	72 ± 2
Heart rate (bpm)	66 ± 2
Biochemical Parameters	
Hemoglobin (g/dL)	14 ± 0.2
Hematocrit (%)	42 ± 0.6
Serum sodium (mmol/L)	139 ± 0.3
Serum potassium (mmol/L)	4.2 ± 0.06
Serum chloride (mmol/L)	104.4 ± 0.34
Plasma osmolality (mOsm/kg H_2_O)	290 ± 0.84
Serum creatinine (mg/dL)	0.90 ± 0.03
Blood urea nitrogen (mg/dL)	13 ± 0.6
Fasting glucose (mg/dL)	87 ± 1.2
Fasting total cholesterol (mg/dL)	163 ± 6.2
Fasting HDL (mg/dL)	67 ± 2.1
Fasting LDL (mg/dL)	90 ± 4.8
Fasting triglycerides (mg/dL)	81 ± 7.8

Values are mean ± SE. BP, blood pressure; HDL, high density lipoprotein; LDL, low density lipoprotein.

**Table 2:** Hemodynamic and renal responses to dietary potassium and sodium perturbation.

	MK/LS	MK/HS	HK/HS
Mass (kg)	70.6 ± 1.8	71.8 ± 1.8 *	71.6 ± 1.9 *
Hemoglobin (g/dL)	13.4 ± 0.4	12.9 ± 0.4	13.05 ± 0.3
Hematocrit (%)	40.7 ± 1.1	39.2 ± 1.1	40.8 ± 1.07
Plasma osmolality (mOsm/kg H_2_O)	287 ± 1.3	289 ± 1	289 ± 1
Serum sodium (mmol/L)	139.7 ± 0.6	140.8 ± 0.5 *	140.1 ± 0.5
Serum potassium (mmol/L)	3.95 ± 0.06	3.99 ± 0.06	4.03 ± 0.05
Serum chloride (mmol/L)	101.7 ± 0.46	103.7 ± 0.37 *	103.1 ± 0.45 *
Urine osmolality (mOsm/kg H_2_O)	324 ± 22.6	465 ± 32.4 *	420 ± 24.3 *
Urine flow rate (mL/min)	1.45 ± 0.1	1.63 ± 0.11	1.72 ± 0.1 *
Free water clearance (mL/min)	0.002 ± 0.09	−0.69 ± 0.12 *	−0.57 ± 0.10 †^,^*
24-h Systolic BP (mm Hg)	116 ± 1	116 ± 1	115 ± 1
24-h Diastolic BP (mm Hg)	70 ± 1	68 ± 1	66 ± 2
24-h PP (mm Hg)	47 ± 1 †	49 ± 1	47 ± 1 †
24-h Heart rate (bpm)	69 ± 2	67 ± 2 *	67 ± 2 *
Laboratory Systolic BP (mm Hg)	110 ± 2	112 ± 2	110 ± 2
Laboratory Diastolic BP (mm Hg)	66 ± 2	66 ± 2	65 ± 2
Laboratory MAP (mmHg)	81 ± 2	81 ± 2	80 ± 2
Laboratory PP (mmHg)	44 ± 2	45 ± 1	44 ± 1

Mean ± SE; BP, blood pressure; HK/HS, high potassium/high sodium; MAP, mean arterial pressure; MK/HS, moderate potassium/high sodium; MK/LS, moderate potassium/low sodium; PP, pulse pressure. * *p* < 0.05 v. MK/LS; † *p* < 0.05 v. MK/HS.

**Table 3:** Vascular measurement responses to dietary potassium and sodium perturbation.

	MK/LS	MK/HS	HK/HS
Brachial artery FMD (mm Δ)	0.23 ± 0.002	0.20 ± 0.002	0.23 ± 0.002
Baseline brachial artery diameter (mm)	3.54 ± 0.01	3.47 ± 0.013	3.57 ± 0.01
Peak brachial artery diameter (mm)	3.77 ± 0.001	3.66 ± 0.01	3.79 ± 0.01
AUC shear rate	27236 ± 3774	28446 ± 3880	33785 ± 4187

Values are mean ± SE. AUC, area under the curve; FMD, flow-mediated dilation; HK/HS, high potassium/high sodium; MK/HS, moderate potassium/high sodium; MK/LS, moderate potassium/low sodium.

## References

[1] Benjamin E.J., Muntner P., Alonso A., Bittencourt M.S., Callaway C.W., Carson A.P., Chamberlain A.M., Chang A.R., Cheng S., Das S.R. (2019). Heart Disease and Stroke Statistics—2019 Update: A Report from the American Heart Association. Circulation.

[2] Sacks F.M., Svetkey L.P., Vollmer W.M., Appel L.J., Bray G.A., Harsha D., Obarzanek E., Conlin P.R., Miller E.R., Simons-Morton D.G. (2001). Effects on blood pressure of reduced dietary sodium and the Dietary Approaches to Stop Hypertension (DASH) diet. DASH-Sodium Collaborative Research Group. N. Engl. J. Med..

[3] Aaron K.J., Sanders P.W. (2013). Role of dietary salt and potassium intake in cardiovascular health and disease: A review of the evidence. Mayo Clin. Proc..

[4] Aburto N.J., Hanson S., Gutierrez H., Hooper L., Elliott P., Cappuccio F.P. (2013). Effect of increased potassium intake on cardiovascular risk factors and disease: Systematic review and meta-analyses. BMJ.

[5] Cook N.R., Obarzanek E., Cutler J.A., Buring J.E., Rexrode K.M., Kumanyika S.K., Appel L.J., Whelton P.K. (2009). Joint effects of sodium and potassium intake on subsequent cardiovascular disease: The Trials of Hypertension Prevention follow-up study. Arch. Intern. Med..

[6] U.S. Department of Health and Human Services and U.S. Department of Agriculture (2015). 2015–2020 Dietary Guidelines for Americans.

[7] National Academies of Sciences, Engineering, and Medicine (2019). Dietary Reference Intakes for Sodium and Potassium.

[8] Luscher T.F., Tanner F.C., Tschudi M.R., Noll G. (1993). Endothelial dysfunction in coronary artery disease. Annu. Rev. Med..

[9] Thijssen D.H.J., Bruno R.M., van Mil A., Holder S.M., Faita F., Greyling A., Zock P.L., Taddei S., Deanfield J.E., Luscher T. (2019). Expert consensus and evidence-based recommendations for the assessment of flow-mediated dilation in humans. Eur. Heart J..

[10] Kido M., Ando K., Onozato M.L., Tojo A., Yoshikawa M., Ogita T., Fujita T. (2008). Protective effect of dietary potassium against vascular injury in salt-sensitive hypertension. Hypertension.

[11] Matsui H., Shimosawa T., Uetake Y., Wang H., Ogura S., Kaneko T., Liu J., Ando K., Fujita T. (2006). Protective effect of potassium against the hypertensive cardiac dysfunction: Association with reactive oxygen species reduction. Hypertension.

[12] DuPont J.J., Greaney J.L., Wenner M.M., Lennon-Edwards S.L., Sanders P.W., Farquhar W.B., Edwards D.G. (2013). High dietary sodium intake impairs endothelium-dependent dilation in healthy salt-resistant humans. J. Hypertens..

[13] Lennon-Edwards S., Ramick M.G., Matthews E.L., Brian M.S., Farquhar W.B., Edwards D.G. (2014). Salt loading has a more deleterious effect on flow-mediated dilation in salt-resistant men than women. Nutr. Metab. Cardiovasc. Dis..

[14] Blanch N., Clifton P.M., Petersen K.S., Keogh J.B. (2015). Effect of sodium and potassium supplementation on vascular and endothelial function: A randomized controlled trial. Am. J. Clin. Nutr..

[15] Schulman I.H., Aranda P., Raij L., Veronesi M., Aranda F.J., Martin R. (2006). Surgical menopause increases salt sensitivity of blood pressure. Hypertension.

[16] Eisenach J.H., Gullixson L.R., Kost S.L., Joyner M.J., Turner S.T., Nicholson W.T. (2012). Sex differences in salt sensitivity to nitric oxide dependent vasodilation in healthy young adults. J. Appl. Physiol..

[17] Hoy M.K., Goldman J.D. (2012). Potassium Intake of the U.S. Population: What We Eat in America, NHANES 2009–2010.

[18] Appel L.J., Baker D., Bar-Or O., Minaker K.L., Morris R.C., Resnick L.M., Sawka M.N., Volpe S.L., Weinberger M.H., Whelton P.K. (2005). Dietary Reference Intakes for Water, Potassium, Sodium, Chloride, and Sulfate.

[19] Frankenfield D., Roth-Yousey L., Compher C. (2005). Comparison of predictive equations for resting metabolic rate in healthy nonobese and obese adults: A systematic review. J. Am. Diet. Assoc..

[20] Goodwin J., Bilous M., Winship S., Finn P., Jones S.C. (2007). Validation of the Oscar 2 oscillometric 24-h ambulatory blood pressure monitor according to the British Hypertension Society protocol. Blood Press. Monit..

[21] Mena L.J., Maestre G.E., Hansen T.W., Thijs L., Liu Y., Boggia J., Li Y., Kikuya M., Bjorklund-Bodegard K., Ohkubo T. (2014). How many measurements are needed to estimate blood pressure variability without loss of prognostic information?. Am. J. Hypertens..

[22] Schmidlin O., Sebastian A.F., Morris R.C. (2007). What initiates the pressor effect of salt in salt-sensitive humans? Observations in normotensive blacks. Hypertension.

[23] Overlack A., Ruppert M., Kolloch R., Gobel B., Kraft K., Diehl J., Schmitt W., Stumpe K.O. (1993). Divergent hemodynamic and hormonal responses to varying salt intake in normotensive subjects. Hypertension.

[24] Sharma A.M., Schattenfroh S., Kribben A., Distler A. (1989). Reliability of salt-sensitivity testing in normotensive subjects. Klin. Wochenschr..

[25] Weinberger M.H. (1996). Salt sensitivity of blood pressure in humans. Hypertension.

[26] Kurtz T.W., DiCarlo S.E., Pravenec M., Morris R.C. (2017). An Appraisal of Methods Recently Recommended for Testing Salt Sensitivity of Blood Pressure. J. Am. Heart Assoc..

[27] Pyke K.E., Tschakovsky M.E. (2007). Peak vs. total reactive hyperemia: Which determines the magnitude of flow-mediated dilation?. J. Appl. Physiol..

[28] Grimm R.H., Neaton J.D., Elmer P.J., Svendsen K.H., Levin J., Segal M., Holland L., Witte L.J., Clearman D.R., Kofron P. (1990). The influence of oral potassium chloride on blood pressure in hypertensive men on a low-sodium diet. N. Engl. J. Med..

[29] Green D.J., Jones H., Thijssen D., Cable N.T., Atkinson G. (2011). Flow-mediated dilation and cardiovascular event prediction: Does nitric oxide matter?. Hypertension.

[30] Choi H.Y., Park H.C., Ha S.K. (2015). Salt Sensitivity and Hypertension: A Paradigm Shift from Kidney Malfunction to Vascular Endothelial Dysfunction. Electrolytes Blood Press..

[31] Fujita T., Ando K. (1984). Hemodynamic and endocrine changes associated with potassium supplementation in sodium-loaded hypertensives. Hypertension.

[32] Rodrigues S.L., Baldo M.P., Machado R.C., Forechi L., Molina Mdel C., Mill J.G. (2014). High potassium intake blunts the effect of elevated sodium intake on blood pressure levels. J. Am. Soc. Hypertens..

[33] Ishimitsu T., Tobian L. (1996). High potassium diets reduce endothelial permeability in stroke-prone spontaneously hypertensive rats. Clin. Exp. Pharmacol. Physiol..

[34] Ying W.Z., Aaron K., Wang P.X., Sanders P.W. (2009). Potassium inhibits dietary salt-induced transforming growth factor-beta production. Hypertension.

[35] Sugimoto T., Tobian L., Ganguli M.C. (1988). High potassium diets protect against dysfunction of endothelial cells in stroke-prone spontaneously hypertensive rats. Hypertension.

[36] Blanch N., Clifton P.M., Petersen K.S., Willoughby S.R., Keogh J.B. (2014). Effect of high potassium diet on endothelial function. Nutr. Metab. Cardiovasc. Dis..

[37] Blanch N., Clifton P.M., Keogh J.B. (2014). Postprandial effects of potassium supplementation on vascular function and blood pressure: A randomised cross-over study. Nutr. Metab. Cardiovasc. Dis..

[38] Oh K.S., Oh Y.T., Kim S.W., Kita T., Kang I., Youn J.H. (2011). Gut sensing of dietary K^+^ intake increases renal K^+^ excretion. Am. J. Physiol. Regul. Integr. Comp. Physiol..

[39] Preston R.A., Afshartous D., Rodco R., Alonso A.B., Garg D. (2015). Evidence for a gastrointestinal-renal kaliuretic signaling axis in humans. Kidney Int..

[40] Baric L., Drenjancevic I., Matic A., Stupin M., Kolar L., Mihaljevic Z., Lenasi H., Seric V., Stupin A. (2019). Seven-Day Salt Loading Impairs Microvascular Endothelium-Dependent Vasodilation without Changes in Blood Pressure, Body Composition and Fluid Status in Healthy Young Humans. Kidney Blood Press. Res..

[41] Graham U.M., McCance D.R., Young I.S., Mullan K.R. (2014). A randomised controlled trial evaluating the effect of potassium supplementation on vascular function and the renin-angiotensin-aldosterone system. J. Hum. Hypertens..

[42] Matthesen S.K., Larsen T., Vase H., Lauridsen T.G., Pedersen E.B. (2012). Effect of potassium supplementation on renal tubular function, ambulatory blood pressure and pulse wave velocity in healthy humans. Scand. J. Clin. Lab. Investig..

[43] Berry S.E., Mulla U.Z., Chowienczyk P.J., Sanders T.A. (2010). Increased potassium intake from fruit and vegetables or supplements does not lower blood pressure or improve vascular function in UK men and women with early hypertension: A randomised controlled trial. Br. J. Nutr..

[44] Lakatta E.G., Levy D. (2003). Arterial and cardiac aging: Major shareholders in cardiovascular disease enterprises: Part II: The aging heart in health: Links to heart disease. Circulation.

[45] Lennon-Edwards S., Allman B.R., Schellhardt T.A., Ferreira C.R., Farquhar W.B., Edwards D.G. (2014). Lower potassium intake is associated with increased wave reflection in young healthy adults. Nutr. J..

[46] He F.J., Marciniak M., Carney C., Markandu N.D., Anand V., Fraser W.D., Dalton R.N., Kaski J.C., MacGregor G.A. (2010). Effects of potassium chloride and potassium bicarbonate on endothelial function, cardiovascular risk factors, and bone turnover in mild hypertensives. Hypertension.

[47] Boegehold M.A. (2013). The effect of high salt intake on endothelial function: Reduced vascular nitric oxide in the absence of hypertension. J. Vasc. Res..

[48] Lenda D.M., Sauls B.A., Boegehold M.A. (2000). Reactive oxygen species may contribute to reduced endothelium-dependent dilation in rats fed high salt. Am. J. Physiol. Heart Circ. Physiol..

[49] Lenda D.M., Boegehold M.A. (2002). Effect of a high-salt diet on oxidant enzyme activity in skeletal muscle microcirculation. Am. J. Physiol. Heart Circ. Physiol..

[50] Nurkiewicz T.R., Boegehold M.A. (2007). High salt intake reduces endothelium-dependent dilation of mouse arterioles via superoxide anion generated from nitric oxide synthase. Am. J. Physiol. Regul. Integr. Comp. Physiol..

[51] Zhu J., Huang T., Lombard J.H. (2007). Effect of high-salt diet on vascular relaxation and oxidative stress in mesenteric resistance arteries. J. Vasc. Res..

[52] Munzel T., Heitzer T., Harrison D.G. (1997). The physiology and pathophysiology of the nitric oxide/superoxide system. Herz.

[53] Greaney J.L., DuPont J.J., Lennon-Edwards S.L., Sanders P.W., Edwards D.G., Farquhar W.B. (2012). Dietary sodium loading impairs microvascular function independent of blood pressure in humans: Role of oxidative stress. J. Physiol..

[54] Ramick M.G., Brian M.S., Matthews E.L., Patik J.C., Seals D.R., Lennon S.L., Farquhar W.B., Edwards D.G. (2019). Apocynin and Tempol ameliorate dietary sodium-induced declines in cutaneous microvascular function in salt-resistant humans. Am. J. Physiol. Heart Circ. Physiol..

[55] Taddei S., Mattei P., Virdis A., Sudano I., Ghiadoni L., Salvetti A. (1994). Effect of potassium on vasodilation to acetylcholine in essential hypertension. Hypertension.

[56] Zicha J., Dobesova Z., Behuliak M., Kunes J., Vaneckova I. (2011). Preventive dietary potassium supplementation in young salt-sensitive Dahl rats attenuates development of salt hypertension by decreasing sympathetic vasoconstriction. Acta Physiol..

